# Laryngeal Imaging Study of Glottal Attack/Offset Time in Adductor Spasmodic Dysphonia during Connected Speech

**DOI:** 10.3390/app13052979

**Published:** 2023-02-25

**Authors:** Maryam Naghibolhosseini, Stephanie R. C. Zacharias, Sarah Zenas, Farrah Levesque, Dimitar D. Deliyski

**Affiliations:** 1 Department of Communicative Sciences and Disorders, Michigan State University, East Lansing, MI 48824, USA; 2 Head and Neck Regenerative Medicine Program, Mayo Clinic, Scottsdale, AZ 85259, USA; 3 Department of Otolaryngology-Head and Neck Surgery, Mayo Clinic, Phoenix, AZ 85054, USA; 4 Lyman Briggs College, Michigan State University, East Lansing, MI 48825, USA; 5 Warrington College of Business, University of Florida, Gainesville, FL 32611, USA

**Keywords:** adductor spasmodic dysphonia, high-speed videoendoscopy, laryngeal imaging, connected speech, glottal attack time, glottal offset time

## Abstract

Adductor spasmodic dysphonia (AdSD) disrupts laryngeal muscle control during speech and, therefore, affects the onset and offset of phonation. In this study, the goal is to use laryngeal high-speed videoendoscopy (HSV) to measure the glottal attack time (GAT) and glottal offset time (GOT) during connected speech for normophonic (vocally normal) and AdSD voices. A monochrome HSV system was used to record readings of six CAPE-V sentences and part of the “Rainbow Passage” from the participants. Three raters visually analyzed the HSV data using a playback software to measure the GAT and GOT. The results show that the GAT was greater in the AdSD group than in the normophonic group; however, the clinical significance of the amount of this difference needs to be studied further. More variability was observed in both GATs and GOTs of the disorder group. Additionally, the GAT and GOT time series were found to be nonstationary for the AdSD group while they were stationary for the normophonic voices. This study shows that the GAT and GOT measures can be potentially used as objective markers to characterize AdSD. The findings will potentially help in the development of standardized measures for voice evaluation and the accurate diagnosis of AdSD.

## 1. Introduction

The use of videostroboscopy in clinical voice assessment is limited in providing enough details about the vocal fold vibrations. This is due to the low frame rate of videostroboscopy, which can only produce an illusory slow-motion of a vibratory cycle [1]. The synchronization of the videostroboscopy to acoustic signals limits its use to capturing the vocal folds vibration only when the vibration is periodic [2,3]. Hence, these limitations become more prominent when studying aperiodic vocal fold vibrations, which is often the case in patients with voice disorders. The use of laryngeal high-speed videoendoscopy (HSV) overcomes the limitations of videostroboscopy with low temporal resolution [4,5]. HSV can provide large amounts of details on the intra-cycle vibratory characteristics of the vocal folds, and it can be used to study dysphonic voices [6–8]. Utilizing HSV in connected speech enables us to study voice disorders in a natural context, where voice disorders reveal themselves [9,10].

Adductor spasmodic dysphonia (AdSD) is a type of focal dystonia that affects the laryngeal muscles and interferes with vocal fold vibrations and voice production. Perceptually, AdSD results in a strained, broken, breathy, and strangled voice [11] and is commonly misdiagnosed with muscle tension dysphonia or vocal tremor [12]. This leads to delayed, ineffective, time-consuming, and expensive treatments, causing obvious patient frustration [12]. Case history, auditory-perceptual voice evaluation, acoustic analysis, and videostroboscopy are widely used for the diagnosis of AdSD and treatment efficacy assessment [13]. A three-tiered approach including a screening questionnaire, speech examination, and nasolaryngoscopy examination was proposed for AdSD diagnosis [14]. Although relatively useful [12,15,16], the diagnosis may still not be certain using these tools including the three-tiered approach [16]. Currently, there are no standardized diagnostic tests for AdSD, and the clinical diagnosis depends on the specialists in the voice care teams [14].

Current literature studying AdSD using HSV has focused on steady-state phonation [17,18]. Patel et al. noted micromotions and oscillatory breaks in AdSD patients when compared to muscle tension dysphonia [17], while Chen and colleagues studied the onset of voicing and found a subset of their AdSD patients presented with differences in the onset phonation gestures [18]. However, AdSD symptoms typically present during connected speech, not sustained phonation. Therefore, studying sustained phonation may limit task-specific characteristics of AdSD. We have previously developed several methods for automated analysis of HSV data during connected speech [9,10,19–24]; however, we still need to conduct manual analysis in order to build new measures and validate our automated methods.

The purpose of the current study is to expand on the above-mentioned studies by obtaining connected speech samples in patients with AdSD and those with no voice disorder (also known as normophonic) voices and quantitatively analyze the beginning and end of each phonation. Toward this goal, the glottal attack time (GAT) and glottal offset time (GOT) were measured for AdSD and normophonic voices in the connected speech samples. The GAT was defined as the time between the first oscillation and the first contact of the vocal folds at the phonation onset [25]. AdSD is characterized by difficulty at the onset of voicing [26]; hence, the GAT would be a suitable measure to capture the abrupt voice initiation [25]. The transitory phonatory phases at the onset and offset of phonation have clinical importance and have been studied during sustained vocalization [27]. The GAT measure was validated previously using electroglottography and acoustic data to characterize the onset of phonation [25]. The GOT was defined as the time difference between the last oscillation and the last contact of the vocal folds [28]. In this study, the GAT and GOT measurement for several participants was conducted by three raters to increase the measurement reliability. The measures were compared and the sources of discrepancies between the raters were determined by two independent evaluators. The inter-rater reliability was assessed and the rater with the most reliable measurements completed the GAT/GOT measurements for the additional participants. Parametric and non-parametric statistical analyses were conducted to compare the GAT and GOT of the normophonic and AdSD groups.

## 2. Materials and Methods

### 2.1. Data Collection

The data were obtained from five normophonic voices (three female and two male) and five with AdSD (four female and one male). HSV data were collected during the reading of the six CAPE-V sentences and the first six sentences of the Rainbow Passage. The HSV system consisted of a Photron FASTCAM mini AX200, M4 (32GB), monochrome high-speed camera (Photron Inc., San Diego, CA, USA), coupled with a flexible nasolaryngoscope. The recordings were conducted at a frame rate of 4000 frames per second (fps) with a spatial resolution of 256 × 224 pixels. HSV recordings of each participant included 200,000–400,000 frames in total and were stored as mraw files (Photron camera format) on an MSI GL63 8SE Laptop (the camera control system) for further analysis.

### 2.2. Participants

The AdSD participants were recruited among the treatment-seeking population at Mayo Clinic, Scottsdale, AZ, diagnosed with AdSD (mean age of 69.2, Table 1). During their initial visits, all participants were seen by both a speech-language pathologist (SLP) and a laryngologist. A voice evaluation and laryngoscopy were conducted during sustained phonation and connected speech, and stimulability tasks were completed. After the full voice evaluation, a diagnosis of AdSD was assigned via multidisciplinary consensus involving a fellowship-trained laryngologist and an SLP, specialized in voice disorders, based on the inclusion criteria (shown in Table 2). The participants with the AdSD diagnosis had no evidence of tremor via consensus between the treating SLP and laryngologist. Participants in the normophonic group were recruited among Mayo Clinic employees and by word of mouth (mean age 47.6, Table 1). All normophonic participants met the inclusion criteria in Table 2. All participants consented to the study. This study was approved by the Institutional Review Board at Mayo Clinic and Michigan State University. All study examinations were carried out by an SLP with 15 years of clinical voice experience.

### 2.3. Data Analysis

The timestamps for the first oscillation, first contact, last oscillation, and last contact of the vocal folds were determined in the HSV data by three raters. The GAT and GOT were then calculated based on the time difference between the oscillation and contact frames. The GATs and GOTs were measured during voice production by the true vocal folds. The oscillations of the false folds were only observed in one subject, i.e., S3M, and accordingly, the GAT and GOT for the false vocal folds were measured for this participant.

The raters visually analyzed the data using playback software (Phantom Camera Control, PCC) with a playback speed of 30 fps. They were allowed to adjust the playback speed as low as 1 fps to select the accurate frame numbers for the contact. The raters did not go below 15 fps for detecting the oscillation frame. The raters applied a Gaussian filter (with a kernel size of 3 × 3) and adjusted the gamma, gain, brightness, and contrast of the frames to facilitate the visual measurements. This filter was used to remove the pattern noise due to the camera lens and endoscope being slightly out of focus. The three raters completed their measurements for four participants and compared their measurements for every vocalization after completing the analysis. To do so, they met over video call and went over the vocalizations for which their measurements were more than 10 frames apart for the contacts and 20 frames apart for the oscillations. Accordingly, the measurements of GAT and GOT by each pair of raters were subtracted from each other (Rater 1-Rater 2, Rater 1-Rater 3, and Rater 2-Rater 3), and the Wald 99% confidence interval was calculated for inter-rater reliability assessment. The raters watched the videos together and came to a consensus about their measurements. Based on this consensus meeting, Rater 3′s measurements were found to be the most reliable measures. Rater 3 improved the accuracy of her measurement based on the consensus meeting and completed the data analysis for the remaining five participants. In this study, Rater 3′s measurements for all participants were used for the data analysis. Additionally, two independent evaluators compared the measurements completed by the three raters to assess the similarity/dissimilarity of measurements among raters and determined the sources of discrepancies. The three Raters reviewed more than 2.6 million HSV images (frames) for the data analysis. Participant N1 was excluded from the analysis due to extremely dark HSV data.

In addition to the descriptive statistics, parametric (ANOVA) and non-parametric tests (Kruskal–Wallis) were used to compare the GAT/GOT between the normophonic and AdSD groups. Moreover, a generalized linear model with repeated observations was used based on the individual measures from the participants. For a regular general linear model (GLM or ANOVA), the estimations are carried out with the covariance matrix forced to sphericity (homoskedasticity); however, this assumption is violated when considering the individual data in this study. Therefore, in this study, rather than using the GLM, the models were built using the PROC MIXED (SAS 9.4) procedure with repeated observations. Instead of the least squares solution, the generalized linear model used a maximum likelihood solution without the sphericity assumption (i.e., the Restricted Maximum Likelihood, REML, solution). In the repeated measures model, additional variables were introduced including gender, age, order of measurements, and interaction terms. At last, the dynamics of the GAT and GOT were evaluated using a time-series analysis.

## 3. Results

The three raters’ measurements for the first and last oscillation, also the first and last contact were within the limits of +/− 18 frames based on the Wald 99% confidence interval. The following is based on what the raters and evaluators concluded as the reasons for their agreement/disagreement with the measurements. More agreement was observed in the measurement of the contact frame number than in the oscillation frame number. More discrepancies in the measurement of the oscillation frame number were found at the offsets of vocalization than at the onsets. The raters were found to be more in agreement when they went further in the analysis of one participant’s data. Generally, more agreement was observed for the measurements of normophonic voices. One of the challenges in the measurement of the disordered voices was determining whether an onset belonged to a new phonation or that there was a delayed onset due to a phonatory break during the same phonation. In the measurements by Rater 1, in several instances, a continuation of phonation after an obstruction of the vocal folds was considered as a new phonation by mistake, which was more apparent for the disordered participants. The partial obstructions of the view of the vocal folds during phonation were found to be the main source of missing measurements by some raters. Both Rater 1 and 2 found Rater 3′s measurements more accurate for the four participants. Rater 3 adjusted her measurements further during the consensus meeting with the other raters. Rater 3 completed the analysis of the remaining participants considering what she had learned during the consensus meeting.

**Figure 1.**
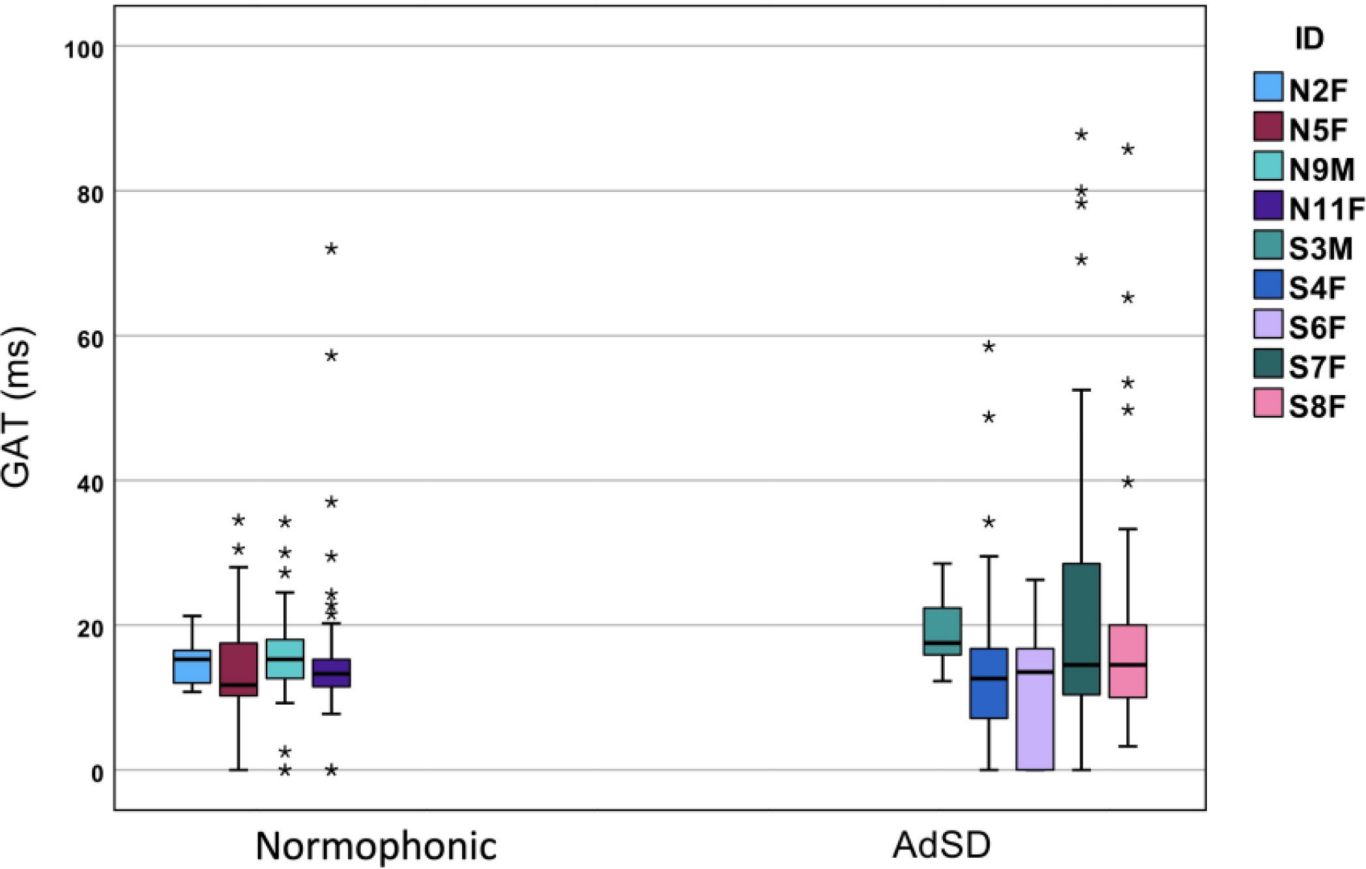
GAT bar graph for normophonic and AdSD voices. The central horizontal line and the bottom and top edges of the boxes indicate the median, 25th, and 75th percentiles, respectively. The whiskers are indicative of extreme data points after the outlier removal. The individual stars show the outliers.

**Figure 2.**
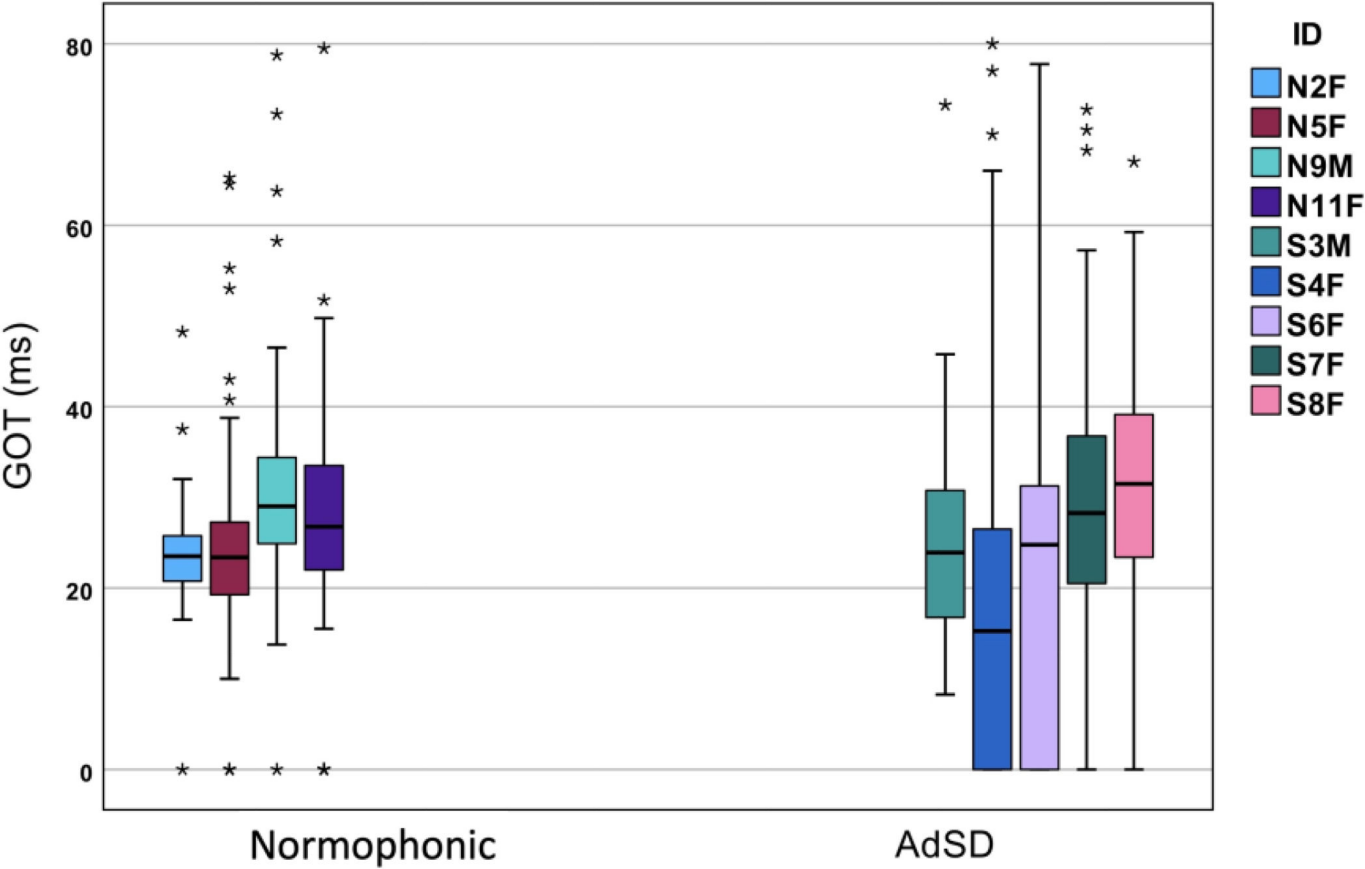
GOT bar graph for normophonic and AdSD voices. The central horizontal line and the bottom and top edges of the boxes indicate the median, 25th, and 75th percentiles, respectively. The whiskers are indicative of extreme data points after the outlier removal. The individual stars show the outliers.

The average GAT in the normophonic group varied from 13.5 to 15.4 ms, and from 11.2 to 22.3 ms for the AdSD group (Figures 1 and 2). The AdSD group showed much greater variability for the GAT compared to the normophonic group: the average coefficient of variability (CV = std/mean x 100) for the AdSD group was 73% compared to 45% for the normophonic group. Greater variability in the AdSD group in comparison with the normophonic group was also observed for the GOTs with an average CV of 70% for the AdSD group vs. 44% for the normophonic group. The AdSD group had higher average GAT values (16.8 ms) than the normophonic group (14.7 ms), but there was no statistically significant difference. The GOT values ranged between 23.7 and 31.5 ms for the normophonic group and between 17.4 and 30.5 ms for the AdSD group. Although the average GOT was slightly higher in the normophonic group (26.4 ms) than in the AdSD group (25.4 ms), no statistically significant difference was observed. The results were confirmed with both parametric tests (ANOVA) and non-parametric tests (Kruskal–Wallis).

We also investigated the relationship between GAT and GOT measures separately for the two groups. Based on the aggregate data, the correlation between the GAT and GOT was very high (Pearson r = 0.635 for the normophonic and r = 0.79 for the AdSD group). The bar graph of the GATs for the normophonic and AdSD group is shown in Figure 1. The data for different subjects are shown with different color bars as indicated in the legend. The median of each participant’s data is shown by the central horizontal lines in the boxes, and the edges of the boxes represent the 25th and 75th percentiles. The whiskers extend to the extreme data points after the outlier removal and the outliers are shown by the individual stars. A similar bar graph for the GOT values for the normophonic and AdSD group is shown in Figure 2. Although more outliers are observed in the AdSD group for the GAT measures (Figure 1), the number of outliers for the GOTs in the normophonic group is higher than in the AdSD group (Figure 2).

The number of onsets/offsets for which the GAT/GOT were measured were compared between the AdSD and normophonic groups. Moreover, the comparison of the number of GAT/GOT with values equal to zero was conducted. None of the differences were statistically significant, but the direction of the effect sizes was as follows: The AdSD group had a lower average number of onsets (42.6 vs. 54) and offsets (50.4 vs. 62.3), a higher number of onsets with zero GATs (4.6 vs. 1.75), and a higher number of offsets with zero GOTs (8.6 vs. 4.8). The statistical tests were repeated after removing the GATs and GOTs with zero values associated with hard glottal attacks/offsets. Accordingly, the AdSD group showed statistically significantly higher average values for GAT than the normophonic group (*p* = 0.037). The GOT averages for the AdSD group were slightly higher than the normophonic group, but the difference was not statistically significant. The results were confirmed with both parametric tests (ANOVA) and non-parametric tests (Kruskal–Wallis).

The generalized linear model with repeated measures showed that the AdSD group had, on average, higher GAT values than the normophonic group (by the average time of 2.4 ms to 4.0 ms), and the difference was statistically significant. The repeated measures model for the GOT did not achieve a clear picture of the effect of the disorder, as it did not show statistically significant differences between the two groups. No effect of gender or age was observed. The GAT and GOT of the normophonic male participants were higher than those of the normophonic females.

**Figure 3.**
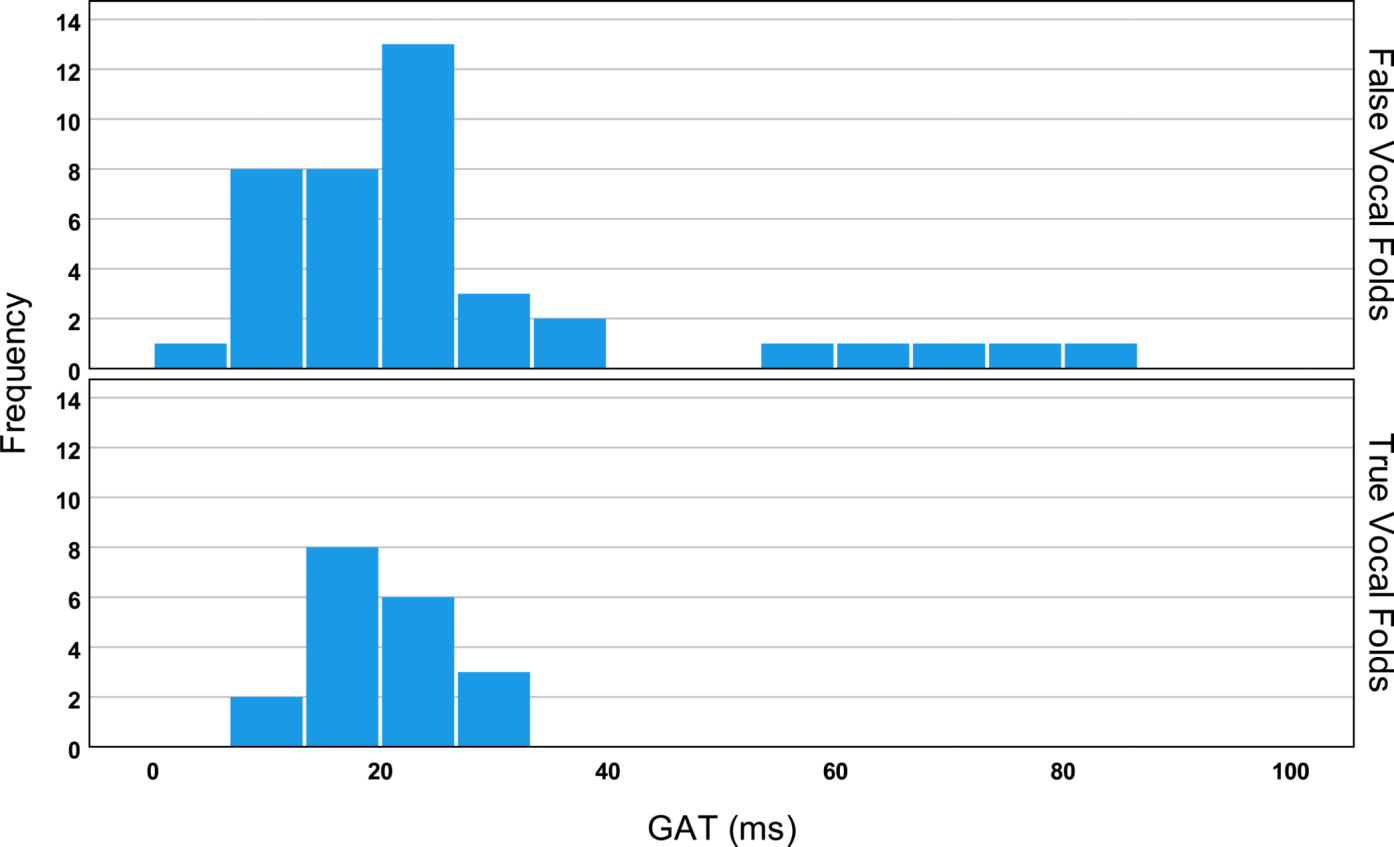
GAT distribution (in ms) for the true vocal folds (**bottom panel**) and false vocal folds (**top panel**).

**Figure 4.**
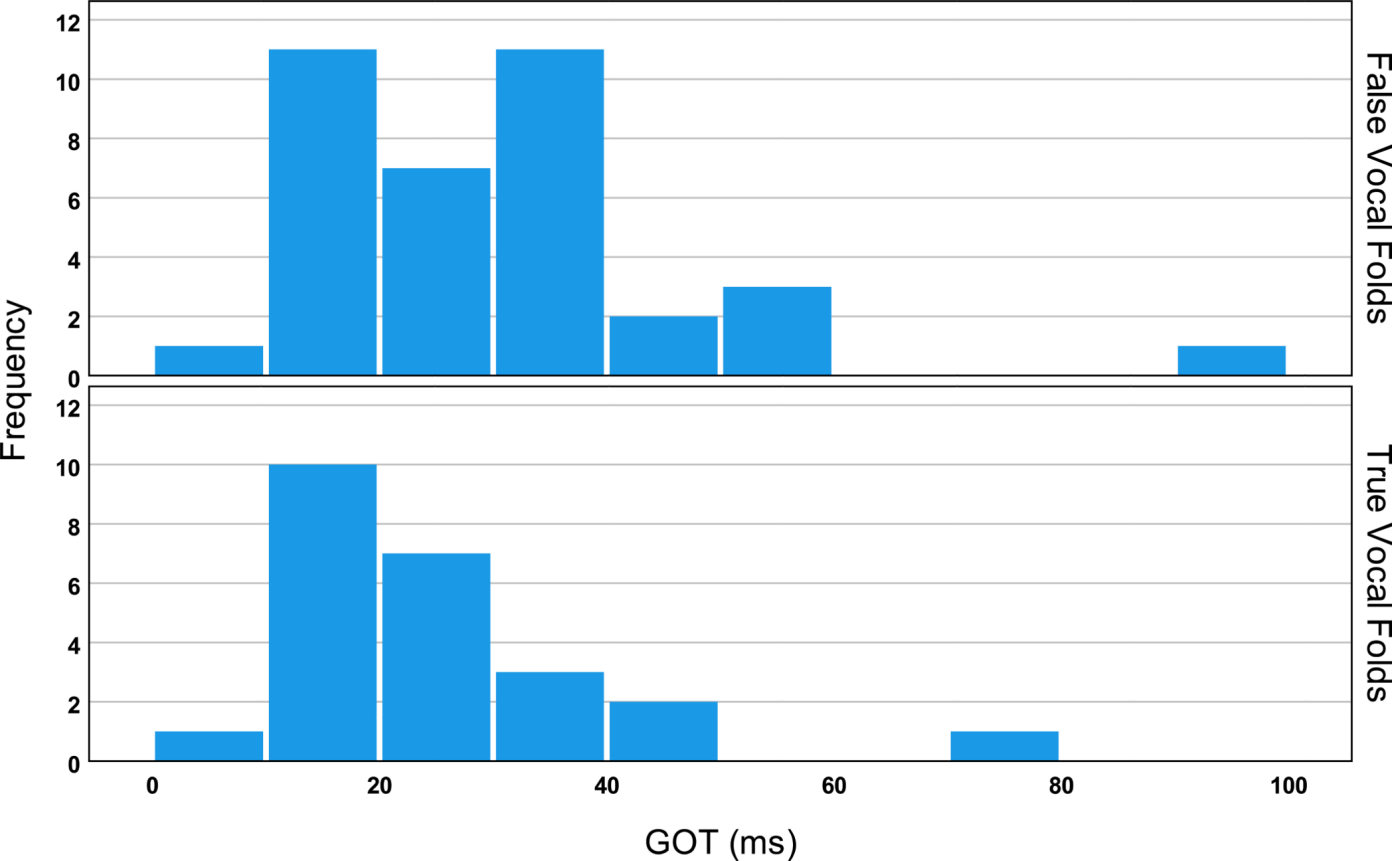
GOT distribution (in ms) for the true vocal folds (**bottom panel**) and false vocal folds (**top panel**).

No statistically significant differences were observed for the GATs/GOTs between the true and false vocal folds onsets/offsets for S3M. However, the average GATs/GOTs of the false vocal folds were longer with higher variation than those of the true vocal folds. The average GAT was 19.4 ms (std of 5.2 ms) for the true vocal folds and 25.9 ms (std of 18.9 ms) for the false vocal folds in S3M. The average GOT was 25.4 ms (std of 14.1 ms) for the true vocal folds and 30.8 ms (std of 17.1 ms) for the false vocal folds. The CV value of 73% for the GATs of the false vocal folds and 27% for the true vocal folds was also indicative of the much greater variability of the GAT of the false vocal folds. The distributions of the GAT and GOT for the true and false vocal folds are shown in Figures 3 and 4, respectively. As can be seen, the GAT/GOT values for the true and false vocal folds overlap; however, greater variability and more extreme values can be observed for the false vocal folds’ measures.

**Figure 5.**
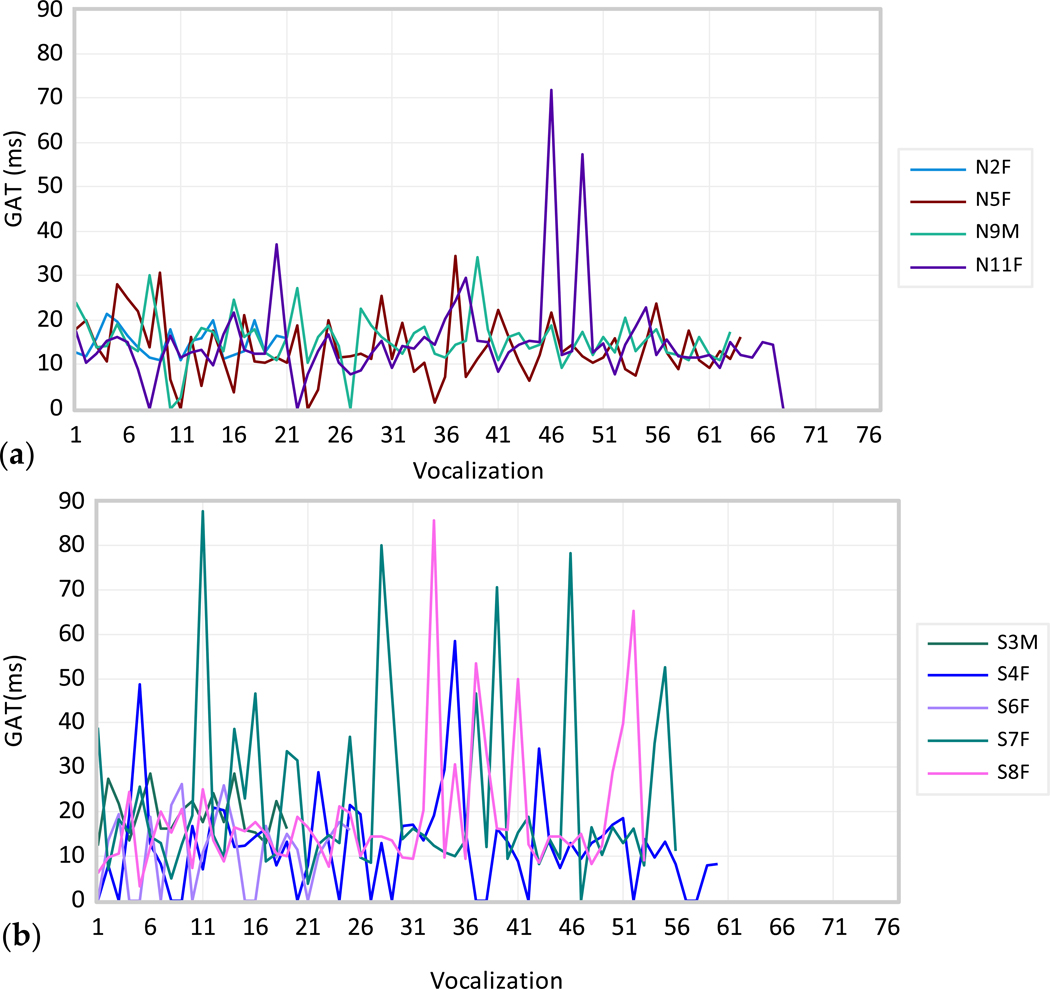
GAT values (in ms) for different vocalizations (*x*-axis) for the normophonic voices (panel (**a**)) and AdSD (panel (**b**)). The subject IDs are shown in the legend.

The dynamics of the individual GAT and GOT time series were analyzed visually in the order they appeared in each participant (see Figure 5). Figure 5 shows the GAT values for different vocalizations for the normophonic group (panel (a)) and AdSD voices (panel (b)). As can be seen in Figure 5a, the dynamics of GAT is clearly a stationary time series for the normophonic group (constant mean across different groups of vocalizations) with homoskedasticity (constant variance across vocalizations). There are only two onsets for participant N11F with larger delays which are outliers. The GAT dynamics for the AdSD group is a nonstationary time series (Figure 5b). As can be seen in Figure 5b, the mean is constantly changing for different groups of vocalizations, and they are periods of small and large variances (heteroscedasticity). The GAT time-series for AdSD shows persistent big swings from one onset to another for almost all patients except for S6F.

Overall, there was a big difference in the dynamics of the GAT time series, representing consecutive onsets, between patients. The contrast between the normophonic and AdSD groups for GOT was slightly different than that of the GAT. The normophonic participants’ GOT dynamics were still stationary but there was a certain large variability (heteroskedasticity). However, the GOT dynamics for the AdSD patients were extremely nonstationary and extremely volatile (constant rapid changes from one offset to another) to a much larger degree than what was observed for the normophonic group.

## 4. Discussion

The results show the GATs are longer in the AdSD group than in the normophonic group, which was confirmed by the generalized linear model analysis. The average GAT was 16.8 and 14.7 ms in the AdSD and normophonic groups, respectively. This was supported by previous research that showed a longer latency between the muscular activation and the phonation onset in AdSD compared to the normophonic voices [29]. Moreover, the steady-state onset delay, the time between the first oscillation and a steady-state oscillation, was observed to be longer in AdSD than in the normophonic voices [18]. While the steady state delay measure is different from the GAT measure in this study, the findings are in line with those of the current work. The GAT values in this study were longer than the GATs measured by Orlikoff et al. [25] for comfortable productions of several sustained vocalizations. The difference between our measurements and those of Orlikoff et al. could be due to the shorter vocal attack times for the selected vowel in their study, while our study measures the GAT for different vocalizations in connected speech. Although the average GOT was slightly shorter in the AdSD group (25.4 vs. 26.4 ms), the parametric and non-parametric analysis did not show any significant differences between the AdSD and normophonic groups. The longer GAT and slightly shorter GOT were also observed in a previous study by our group with a smaller sample size and a different data collection HSV system [28].

A greater variability was observed in both the GAT and GOT in the AdSD group, which was expected as the disorder led to more uncertain behaviors and irregularity in the vibrations of the vocal folds. Considering that the AdSD patients have less neurological control over their laryngeal muscles during voice production [30–32], the longer GAT and the higher variability in the GAT and GOT of AdSD can be explained. The AdSD patients showed nonstationary vocal fold dynamics with large variations in the GAT and even larger ones for the GOT. The dynamics of vocal fold vibration in the normophonic patients were stationary with small variations in the GAT and larger variations in the GOT. The more volatile behavior of the GOT in the normophonic group than that of the GAT was also observed through the larger number of outliers in GOTs. Therefore, GAT might be a more valuable measure than the GOT since it was found to be statistically different between the normophonic and AdSD groups and a more stable measure within the normophonic group. The high correlations between the GAT and GOT values in both groups indicate that the participants with greater GAT values also tend to have greater GOTs and vice versa. A larger number of onsets and offsets with zero GAT and GOT was observed in the AdSD group which could be related to the disorder. Therefore, the number of the hard glottal attack/offset could be potentially used as another measure to characterize the AdSD. The AdSD group also showed a lower average number of onsets and offsets with measurable GATs/GOTs. This was due to the more excessive laryngeal maneuvers in connected speech in the AdSD group that led to more blockage of the view of the vocal folds and a smaller number of measurable GATs/GOTs. No effect of sex and age was observed using the generalized linear model, which could be due to the small sample size in this study.

The comparison of GAT/GOT measured for the true and false vocal folds in S3M showed some overlap. As the false vocal folds’ vibration is a compensatory strategy, more variability and extreme values were observed for GATs/GOTs of the false vocal folds. The GAT and GOT values for the false vocal folds in S3M were the highest among the AdSD group. Although S3M was a male, and this observation might be explained by the lower fundamental frequency of the voice, a similar observation was not made for the GAT/GOT values of the true vocal folds in this participant. Therefore, the longer GAT/GOT of the false vocal folds could be related to the vibratory characteristics of these folds, which will be further investigated in the future.

The inter-rater reliability assessment showed that the measurements by the three raters were reliable as the upper limit of the 99% confidence interval was less than 18 frames. This frame number (criterion) was considered acceptable in the current study since the fundamental frequency of the voices ranged between 100 and 250 Hz. Some discrepancies between the measurements among the raters were expected due to the nature of the data and the manual measurement. Since the glottal closure can last for several frames, it was possible for the raters to select a slightly different contact frame number. Indicating the first/last oscillation was also challenging since the raters needed to select the frame while the video was being played, and, depending on when the video was stopped, the selected frame could have been different. Additionally, due to the small amplitudes of oscillations, more discrepancies between the oscillation frame numbers by the three raters in comparison with the contact frame numbers were expected. These discrepancies were more noticeable at the offset of phonation due to the longer GOTs than the GATs and more (difficult to detect) miniature oscillations at the offset. The software settings, the computers, and the room brightness were different among the raters, which could have been another source for some of the discrepancies. As the raters became more comfortable with the anatomy/physiology of vocal folds, their measurements became more reliable.

Although the observations of the GAT and GOT in this study are in line with our understanding of the AdSD, the dynamic nature of the GAT and GOT measurements in the HSV data limits the accuracy of manual measurement. The detection of the oscillation/contact frames depended on the quality of the data, the image adjustments by the raters, and the visual acuity of the raters. Additionally, the playback speed of the video and the small amplitudes of the vibrations impacted the accurate measurement of the oscillation frames. These challenges became more noticeable when analyzing the AdSD data due to the higher irregularity, unexpected behavior in the vocal fold vibration, and occurrence of phonatory breaks. In future, an automated method will be developed to overcome the challenges of manual measurements. A more detailed comparison of the GAT/GOT values for equivalent vocalizations needs to be performed to provide more accurate assessments of GAT/GOT differences between the normophonic and AdSD voices, which is among our future goals. Additionally, the relationship between GAT/GOT measures and the severity of AdSD, measured by perceptual voice assessment, will be studied. These works are important for indicating the clinical significance of these measures, as the statistical significance alone is not sufficient to interpret the findings of the study [33]. The small sample size of the current study made it difficult to draw conclusions about the impact of sex or age on the measurements. More data are being collected to address the limitations of this study due to the small sample size.

## Figures and Tables

**Table 1.** The characteristics of participants in the normophonic and AdSD groups.

Non-Pathological	Sex	Age					
N1M	M	35					
N2F	F	35					
N5F	F	67					
N9M	M	49					
N11F	F	52					
AdSD (No Tremor)	Sex	Age	Last Injection (months)	Length of Time Botox Lasts (months)	Length of dx (years)	CAPE-V Overall Severity	CAPE-V Most Severe Characteristic
S3M	M	76	3.5	2.5	7	Severe	Strain
* S4F	F	67	5	2	2+	Moderate- Severe	Strain
S6F	F	67	3	2	3	Moderate	Strain
S7F	F	60	4	2	4	N/A	N/A
* S8F	F	76	5	5	5+	Moderate	Strain

* Patient was seen at an outside institution prior to their initial visit to Mayo Clinic, so the exact date of diagnosis is unknown.

**Table 2.** Study inclusion criteria.

Inclusion Criteria	AdSD Group	Normophonic Group
At least 18 years of age and proficiency in reading English written text.	X	X
No prior history of intubation injury or airway/laryngeal surgery.	X	X
Normal hearing.	X	X
Absence of structural abnormalities including lesions and/or vocal fold paralysis/paresis.	X	X
Absence of perceptual symptoms of classical dysarthria.	X	X
Cognitively intact and able to undergo the flexible HSV protocol.	X	X
Auditory-perceptual characteristics consistent with the disorder (evidence of phonatory breaks on voiced sounds and a strained-strangled quality, and no obvious tremor during phonation).	X	
Occasional moments of normal-sounding voice.	X	
Improved voice for non-speech vocalizations.	X	
Improved voice quality for phonation at higher pitches.	X	
Not stimulable for voice change with facilitation techniques (e.g., distraction, improving breath and voice coordination and forward resonance, manual manipulation).	X	
No recent Botox treatment or surgical treatment for AdSD.	X	

## Data Availability

Data from this study can be made available upon request to the corresponding author after executing appropriate data-sharing agreements.
